# Facile Synthesis of Novel Magnetic Janus Graphene Oxide for Efficient and Recyclable Demulsification of Crude Oil-in-Water Emulsion

**DOI:** 10.3390/molecules29143307

**Published:** 2024-07-13

**Authors:** Yingbiao Xu, Li Cheng, Yefei Wang, Han Jia

**Affiliations:** 1 Key Laboratory of Unconventional Oil & Gas Development, China University of Petroleum (East China), Ministry of Education, Qingdao 266580, China; xuyingbiao.slyt@sinopec.com (Y.X.); jiahan@upc.edu.cn (H.J.); 2 Technology Inspection Center, Shengli Oilfield Company, SINOPEC, Dongying 257000, China; 3 School of Petroleum Engineering, Yangtze University, Wuhan 430100, China; chengli_whu@163.com

**Keywords:** crude oil-in-water emulsion, Fe_3_O_4_, magnetic demulsifier, recyclable demulsification, Janus graphene oxide

## Abstract

Nanoparticles have been widely applied to treat emulsion-containing wastewater in the form of chemical demulsifiers, such as SiO_2_, Fe_3_O_4_, and graphene oxide (GO). Owing to their asymmetric structures and selective adsorption, Janus nanoparticles show greater application potential in many fields. In the present work, the novel magnetic Janus graphene oxide (MJGO) nanoparticle was successfully prepared by grafting magnetic Fe_3_O_4_ to the surface of the JGO, and its demulsifying ability to treat a crude oil-in-water emulsion was evaluated. The MJGO structure and its magnetic intensity were verified by Fourier-transform infrared spectra (FTIR), transmission electron microscopy (TEM), X-ray diffraction (XRD), and magnetization saturation (MS) tests. Compared with GO and JGO, MJGO displayed the superior efficiency (>96%) to demulsify the crude oil-in-water emulsion, which can be attributed to the reduced electrostatic repulsion between MJGO and the emulsion droplets. Furthermore, the effects of pH and temperature on the demulsification performance of MJGO were also studied. Lastly, the recyclability of MJGO largely reduced the cost of demulsifiers in separating crude oil and water. The current research presents an efficient and recyclable demulsifier, which provides a new perspective for the structural design of nanomaterials and their application in the field of demulsification.

## 1. Introduction

Treating wastewater produced from oil production is an urgent problem to be solved in the petroleum industry [1,2]. The excessive exploitation has led to the transition of many oilfields into the high water-cut stage, followed by the generation of massive amounts of wastewater containing crude oil-in-water emulsion. The direct discharge of the emulsion-containing wastewater would cause irreversible and permanent damage to the local ecosystem. Therefore, various demulsification methods for separating the crude oil from the emulsion have been studied in depth [3,4,5].

Currently, the proposed methods mainly include gravity separation, membrane separation, adsorption, and flotation [6,7,8]. The chemical demulsifiers possess the advantages of a modifiable structure, cost-effectiveness, and high efficiency in demulsification, and as such they are extensively employed. Additionally, a plethora of demulsifiers have been studied over the past few years, such as polysiloxane, block copolymer, dendrimer, and nanomaterials. In particular, nanomaterials with a high adsorption ability have received much attention for their remarkable demulsification behaviors, such as SiO_2_, Fe_3_O_4_, and graphene oxide (GO) [9,10]. For example, Xie et al. employed chemically modified, fumed silica nanoparticles with different hydrophobicity to separate the prepared emulsion. They proposed that the average hydrophobicity of the nanoparticles determined the demulsification efficiency [11].

GO possesses unique physical−chemical properties, such as a great adsorption capacity, facile modification, and high specific surface area, which endows it with great potential in treating oily wastewater [12,13,14,15]. Liu et al. employed GO nanosheets as a versatile demulsifier to cause the rapid coalescence of the small droplets in the oil-in-water emulsion within a few minutes [16]. Luo et al. introduced an alkyl chain to a single side of GO to prepare Janus GO (JGO) nanosheets with improved amphiphilicity [17]. Jia et al. confirmed the excellent demulsification performance of JGO and achieved efficient separation of heavy oil-in-water emulsions [18,19,20]. However, the non-recyclability of the JGO dramatically increases the cost of separating an emulsion and the risk of environmental pollution. It is urgently necessary to develop a recyclable JGO nanomaterial to treat emulsion-containing wastewater more efficiently.

Fe_3_O_4_ nanoparticles with strong magnetism have been noticed for their recyclable use [21,22,23]. Xu et al. synthesized an Fe_3_O_4_-based magnetic nanoparticle, which exhibited a considerable demulsification performance [24]. More importantly, the prepared nanoparticle could be reused after collecting and washing it. Chen et al. proposed a magnetically recoverable, efficient demulsifier by coating Fe_3_O_4_ with amorphous SiO_2_ [25]. The additional demulsifier efficiently separated the emulsion droplets and could be easily recovered without any decline in efficiency after recycling owing to their paramagnetic properties. However, there are scarce reports about the modification of JGO by Fe_3_O_4_ nanoparticles for the treatment of emulsion-containing wastewater.

In this study, we synthesized the novel recyclable nanomaterial demulsifier MJGO by grafting magnetic Fe_3_O_4_ to the surface of JGO, which possessed both amphiphilicity and magnetism. The successful synthesis was reflected by TEM, FTIR, and XRD. Then, the demulsification performance of the MJGO and its influencing factors (shaking times, dosage, pH, and temperature), as well as the demulsification mechanism, were systematically studied. Finally, the recyclable performance of the MJGO was tested.

## 2. Results and Discussion

### 2.1. Characterization of MJGO

**Figure 1:**
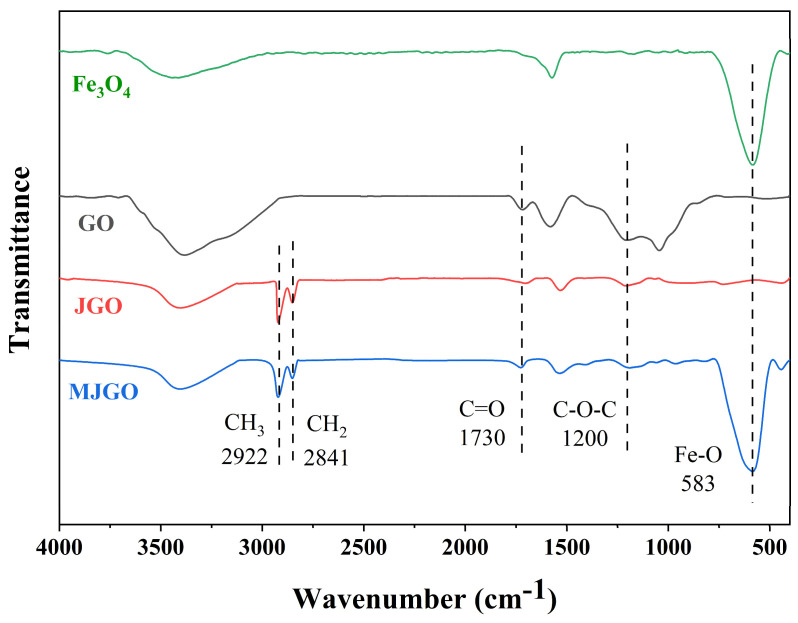
FTIR of Fe_3_O_4_, GO, JGO, and MJGO.

The FTIR of unmodified and modified GO were recorded to demonstrate the successful preparation of the MJGO nanomaterials (Figure 1). The unique peaks at 2922 and 2841 cm^−1^ for the JGO and MJGO samples are assigned to the stretching vibration of C-H (-CH_2_ and -CH_3_), indicating the existence of a hydrocarbon chain [26,27]. Moreover, the strong peak that appears at 583 cm^−1^ is ascribed to the stretch of the Fe-O bond, which reflects that the Fe_3_O_4_ nanoparticles are successfully grafted to the JGO surface [28,29].

**Figure 2:**
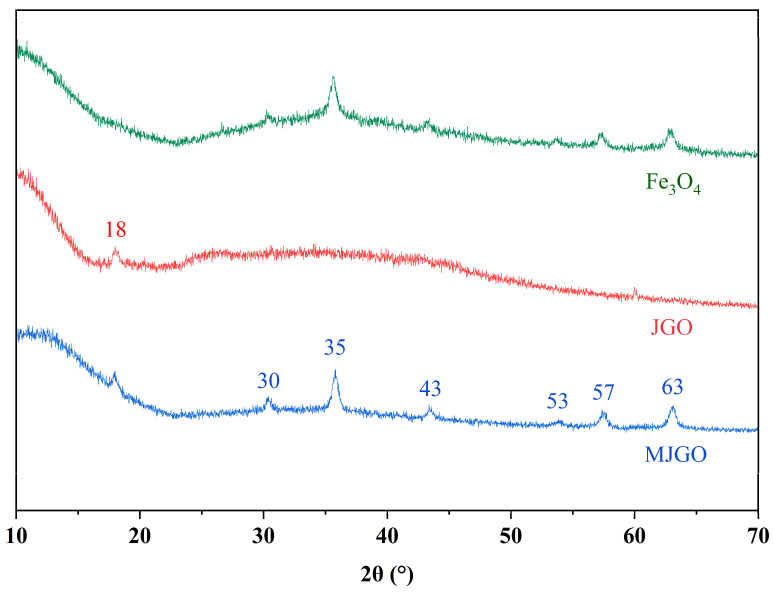
XRD patterns of Fe_3_O_4_, JGO, and MJGO.

The phase composition and crystal structures of the MJGO were characterized by XRD (Figure 2). The presence of Fe_3_O_4_ in the MJGO is confirmed by comparing these peaks with standard peaks (JCPDS: 19-0629) [30]. The XRD pattern of JGO shows an obvious characteristic diffraction peak at 2*θ* = 18°. The diffraction peaks at 30°, 35°, 43°, 53°, 57°, and 63° correspond to (220), (311), (400), (422), (511), and (533) in the crystalline structure of Fe_3_O_4_, respectively [31]. Moreover, the crystallographic properties of the MJGO particles, obtained via Equations (1) and (2), are also shown in Table 1.

(1) $$D=\frac{k\lambda }{\beta cos\theta }$$

(2) nλ=2dsin⁡θ

where *D*, *k*, *λ*, *β*, *θ*, and *d* represent the crystal size (nm), constant dimension shape factor (0.89), wavelength of the incident X-ray (0.1542 nm), full width of the half maximum (FWHM) (radiant), Bragg angle, and interplanar d-spacing (Å), respectively [3]. The crystalline size of the Fe_3_O_4_ nanoparticles attached to the MJGO as calculated from the width of the highest diffraction line (311) via the Debye−Scherrer equation is approximately 27 nm [3].

**Figure 3:**
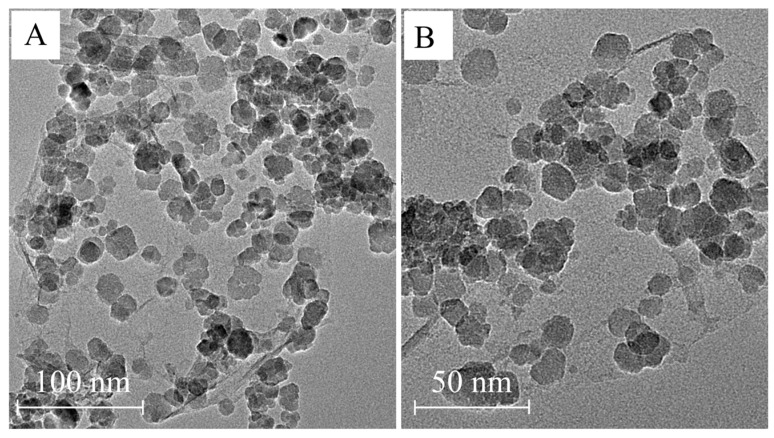
TEM images of MJGO at different magnifications: (**A**) low magnification; (**B**) high magnification.

The TEM images with low and high magnifications directly prove the successful synthesis of the MJGO nanomaterials (Figure 3). It was found that most Fe_3_O_4_ nanoparticles distribute on the JGO surface, and the diameters of the monodispersed Fe_3_O_4_ nanoparticles are about 15–25 nm, which agrees with the size shown in the XRD. The SEM images captured at a high magnification of the MJGO are shown in Figure S1. The amphiphilicity of MJGO causes its spontaneous aggregation. It is evident that the surface of the MJGO’s hydrophilic side is comprehensively covered by the grafted magnetic Fe_3_O_4_ nanoparticles, with an approximate size of 20 nm, which is consistent with the results of the XRD and TEM. However, there is almost no adhesion of Fe_3_O_4_ nanoparticles on the hydrophobic side.

**Figure 4:**
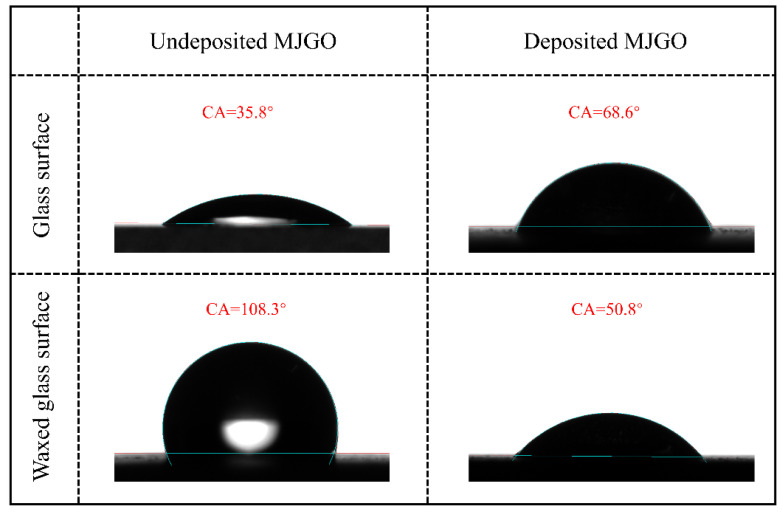
The water contact angles of undeposited MJGO and deposited MJGO glass surfaces and waxed glass surfaces.

In addition, the MJGO exhibits the characteristic ability to reverse wettability due to its amphiphilicity. As depicted in Figure 4, the glass surface exhibits hydrophilicity, while the waxed glass surface displays hydrophobicity, with corresponding water contact angles (CA) of 35.8° and 108.3°, respectively. Then, the deposition of the MJGO leads to the evidently increased CA of the glass surface (68.6°) and simultaneously causes the decreased CA of the wax-coated glass surface to 50.8°. This phenomenon should be attributed to the selective adsorption of the MJGO on both the glass surface and the waxed glass surface. When MJGO is deposited on the hydrophilic glass surface, the hydrophilic side is adsorbed on the glass surface and the hydrophobic alkyl chain towards an outward direction, thereby enhancing the hydrophobicity of the glass surface. Similarly, the hydrophobic side of the MJGO preferentially interacts with the waxed glass surface, and the exposed hydrophilic side results in the transition from hydrophobicity to hydrophilicity.

**Figure 5:**
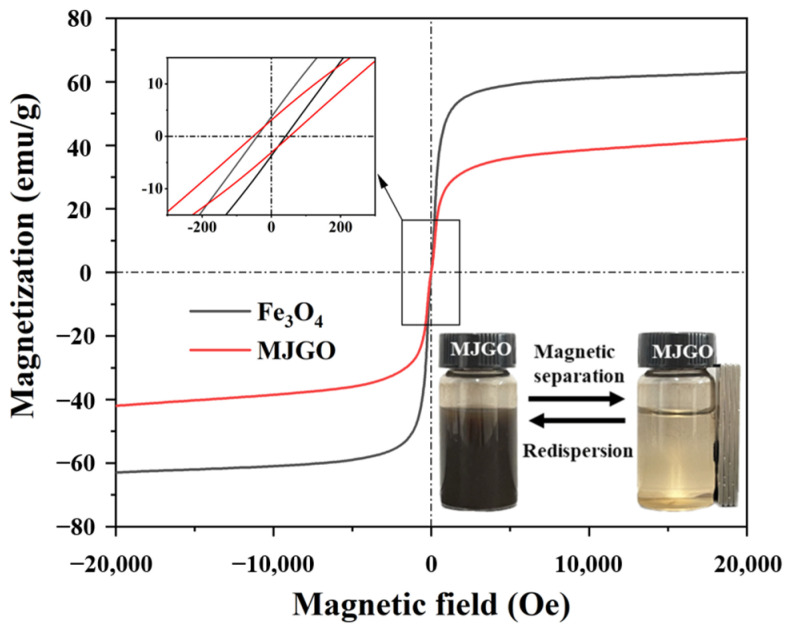
Magnetization curves of Fe_3_O_4_ and MJGO at 298 K.

Figure 5 presents the magnetization curves of Fe_3_O_4_ and MJGO. It is evident that the magnetization of the nanoparticles increases with an enhanced external magnetic field. Compared with the magnetization saturation (MS) of raw Fe_3_O_4_ (62.4 emu/g), the existence of JGO moderately decreases the MS of MJGO [32,33]. Both the Fe_3_O_4_ and MJGO nanomaterials exhibit negligible remanence, suggesting their superparamagnetic property. Meanwhile, the MJGO is well dispersed in water and exhibits great magnetic responsiveness, which can be quickly collected under the action of the external magnetic field.

### 2.2. Demulsification Efficiency Tests of MJGO

**Figure 6:**
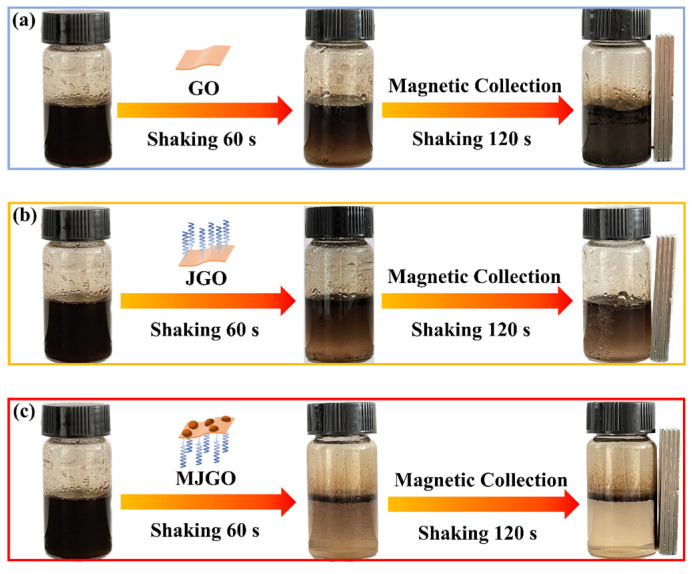
Photographs of a crude oil-in-water emulsion separated by different particles, (**a**) GO, (**b**) JGO, and (**c**) MJGO.

The crude oil-in-water emulsion without additional nanoparticles can remain stable for at least 72 h (Figure S2). When the GO nanoparticles are added into the emulsion, there is no significant change in the emulsion color after it is shaken violently for another 120 s (Figure 6a). The hydrophilic GO nanoparticles show poor interaction with the active substance of crude oil in the present system. Then, the GO nanoparticles hardly achieve the effective demulsification. However, the additional JGO nanoparticles cause evident changes in the crude oil-in-water emulsion color from black to brown, revealing their greater demulsifying ability. After vigorous shaking for 120 s, the emulsion remains visibly opaque, indicating that the JGO nanoparticles exhibit limited efficacy in achieving complete oil−water separation (Figure 6b). Compared with the GO nanoparticles, the much more intensive π–π/n–π interaction between JGO and the active substance of crude oil results in more effective demulsification [34]. However, the non-recyclable JGO nanoparticles used in demulsifying behavior dramatically increase the cost of collecting oil droplets in a crude oil-in-water emulsion. Interestingly, the crude oil-in-water emulsion flocculates rapidly with additional MJGO nanoparticles, and the dark brown emulsion becomes a clear and transparent aqueous solution (Figure 6c). Notably, the absence of oil droplets in the collected water (Figure S3) highlights the remarkable demulsifying efficacy exhibited by MJGO nanoparticles. More importantly, the MJGO nanoparticles can be recycled via magnetic force, which provides numerous potentials for future applications.

**Figure 7:**
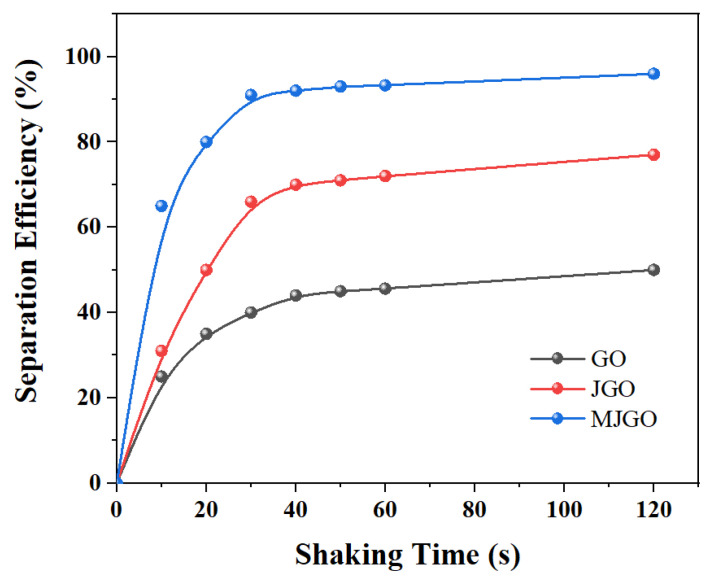
The effects of the shaking time on the separation performance of GO, JGO, and MJGO.

Then, we systematically studied the possible influencing factors for the demulsifying behavior of GO, JGO, and MJGO nanoparticles. As shown in Figure 7, following 40 s of shaking, the emulsion exhibited conspicuous demulsification and stratification, with the processes reaching equilibrium after 120 s. The separation efficiency of the GO nanoparticles was the lowest (~50%), and the demulsification ability of the JGO improved to around 70%. The MJGO nanoparticles exhibited the best demulsification ability, with more than 90% separation efficiency. The result of this test is highly in accordance with the above photographs in Figure 6.

**Figure 8:**
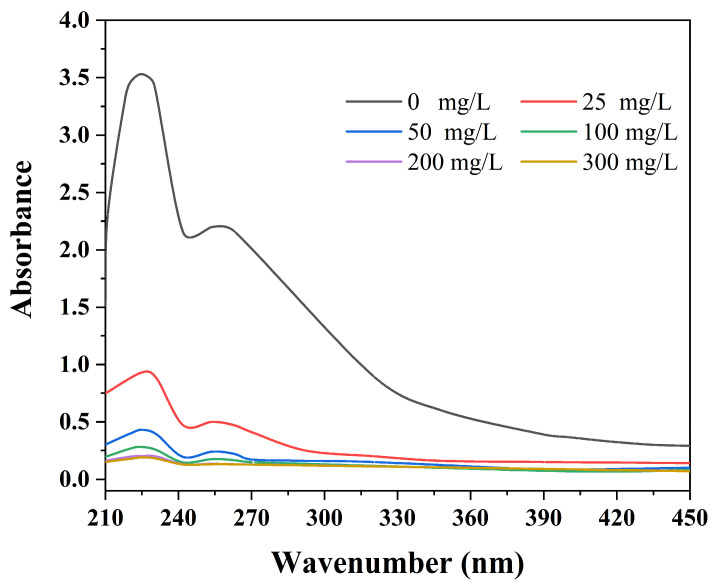
The effects of MJGO concentration on the amount of residual crude oil after demulsification.

To quantitatively explore the effects of the MJGO concentration on its demulsification efficiency, the absorbance of the oil in the separated water was tested via the UV-vis spectrum (Figure 8). The increased concentration of the MJGO resulted in a decrease in the oil’s absorbance, indicating an enhancement in the separation efficiency. The MJGO displayed a remarkable ability in separating oil, even at a much lower concentration (25 mg/L). And the absorbance of MJGO hardly decreased when its concentration increased from 200 mg/L to 300 mg/L. Therefore, 200 mg/L is regarded as the most efficient concentration for MJGO as a demulsifier.

### 2.3. Demulsification Mechanism of MJGO

**Figure 9:**
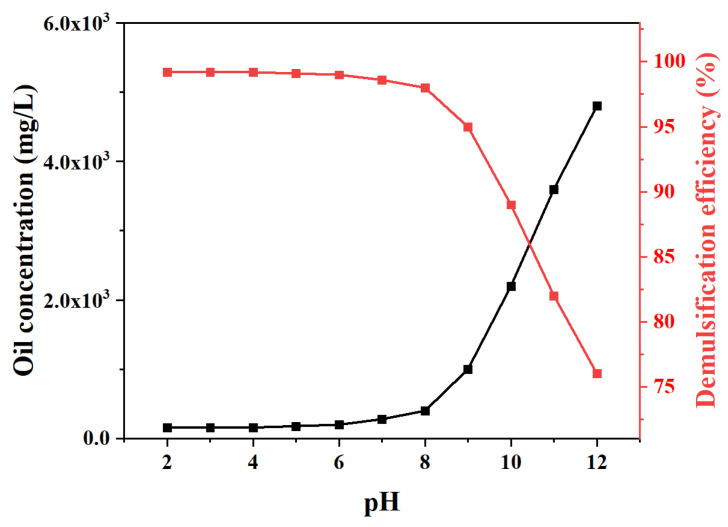
The effects of pH on the demulsification performance of MJGO (200 mg/L).

**Figure 10:**
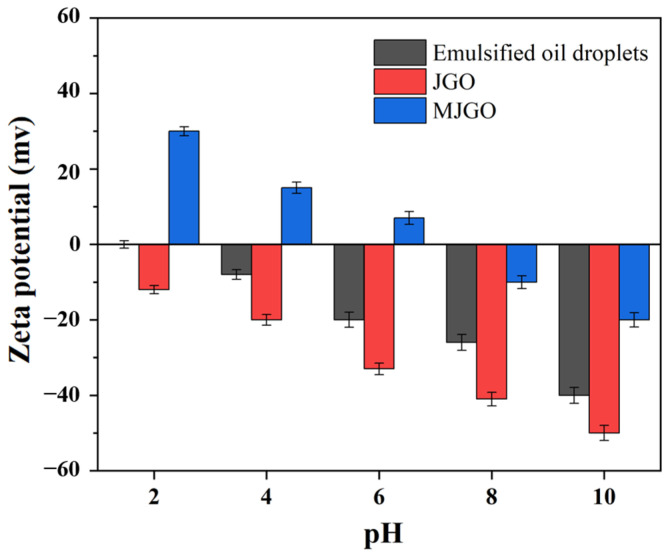
Zeta potential of emulsified oil droplets, JGO dispersion, and MJGO dispersion.

**Figure 11:**
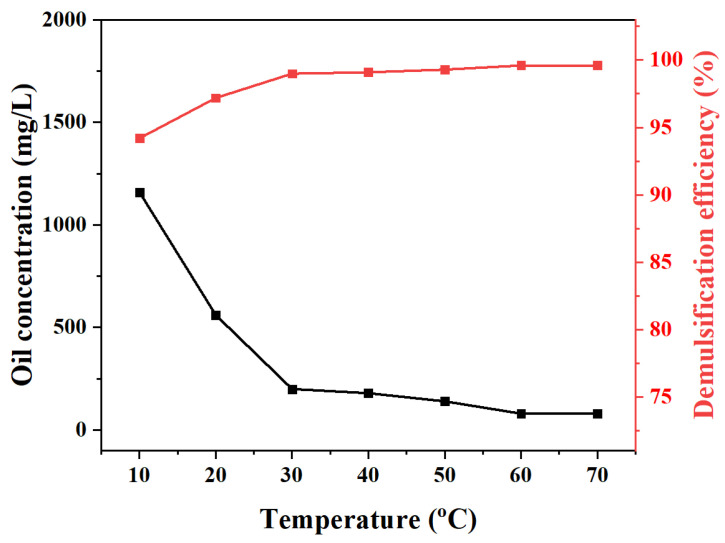
The effects of temperature on the demulsification performance of MJGO (200 mg/L).

The effects of pH and temperature were systematically investigated in the control experiments to explore the demulsification mechanism of the MJGO (Figure 9, Figure 10 and Figure 11). Previous studies have demonstrated that variations in pH and temperature exert a profound influence on the physicochemical characteristics of the demulsifier surface, thereby significantly impacting its efficacy in demulsification [35,36]. Clearly, the demulsification efficiency of MJGO is excellent under acidic and neutral conditions, while its performance is significantly weakened in the alkaline environment (Figure 9). This phenomenon occurs due to the conversion of the organic acid in crude oil into a surface-active material under alkaline conditions, thereby enhancing the emulsion’s stability. In addition, the pH value affects the zeta potential of the MJGO and emulsion droplets, which is another reason for the change in demulsification efficiency (Figure 10). The MJGO is positively charged with the zeta potentials of 30 mV, 15 mV, and 7 mV at the pH values of 2, 4, and 6, respectively. And it further decreases to a negative charge of −10 mV and −20 mV as the pH increases to 8 and 10, respectively. In addition, the increased pH value continually decreases the negative zeta potentials of the emulsified oil droplets from 0 to −41 mV. The elevation of the pH value amplifies the negative charge of the MJGO and the emulsion droplets, thereby augmenting the electrostatic repulsion between them, resulting in a reduction in demulsification efficiency under alkaline conditions (pH ≥ 8). Furthermore, the demulsification performance of the MJGO improves slightly as the temperature increases (Figure 11). The increased temperature accelerates the thermal motion of the emulsion droplets, which is beneficial to the combination with the MJGO. In summary, MJGO demonstrates remarkable efficacy in the separation of crude oil from oil-in-water emulsions across a broad spectrum of temperatures.

In a previous report, JGO displayed considerable demulsifying ability, and its possible demulsification mechanism was proposed [34]. The active substance in crude oil (mainly asphaltene) generally forms a protective film at the interface of oil and water to stabilize the emulsion. The additional JGO can adsorb at the interface and establish interactions with asphaltene. Then, the original interfacial film will be destroyed due to the strong interaction between the JGO and the asphaltene molecules, leading to the coalescence of emulsion droplets. Compared with the JGO, the MJGO possesses lower electronegativity due to the introduction of positively charged Fe_3_O_4_ (Figure 10), which effectively reduces the electrostatic repulsion between the MJGO and the negatively charged oil droplets. Thus, the more intensive adsorption capacity of MJGO should be responsible for its greater performance in demulsification. Moreover, the extra magnetic force further improves the separation between oil and water caused by MJGO with its unique magnetic properties.

### 2.4. Recycling Tests of MJGO

**Figure 12:**
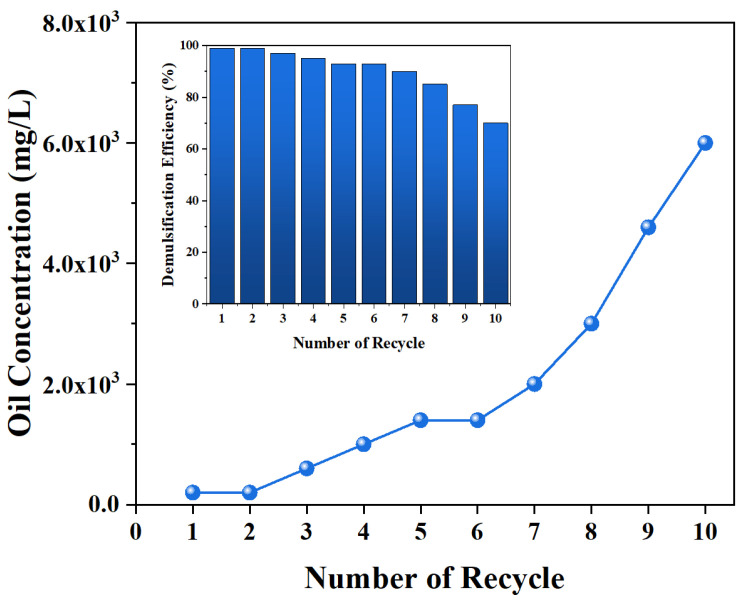
The recycling tests of MJGO.

A conventional chemical demulsifier is limited to single use, whereas magnetic MJGO possesses the distinct advantages of recyclability and reusability. Recycling tests are conducted to assess the potential reusability of the MJGO. Given the aforementioned demulsification mechanism, it is speculated that a significant amount of asphaltenes would undergo adsorption on the MJGO’s surface in the demulsification process [34]. Therefore, the recycled MJGO should be subjected to a toluene wash in order to effectively eliminate the adsorbed oil, particularly the asphaltenes. The oil concentration in the separated water and demulsification efficiency for each cycle are shown in Figure 12. It is found that the oil concentration in the separated water phase is less than 20 mg/L, with the corresponding demulsification efficiency exceeding 90% during the initial 7 cycles. However, the demulsification performance obviously decreases from the eighth cycle. Notably, during the last two cycles, the oil concentration escalates to exceed 5000 mg/L while concomitantly witnessing a decline in demulsification efficiency to approximately 77%. The degradation of the demulsification performance of the MJGO is mainly attributed to the increased intensity of the adsorption of the natural surfactants (asphaltenes and resins) at the MJGO surfaces, which is not completely removed by toluene washing. This results in a significant increase in the absolute zeta potential of the MJGO, particularly after it has been recycled multiple times (Figure S4). In summary, the MJGO exhibits exceptional demulsification performance and remarkable recyclability, which can be efficiently retrieved from intricate multiphase systems through the application of the magnetic field.

## 3. Materials and Methods

### 3.1. Materials

N-octylamine (>98%), ammonium iron (II) sulfate hexahydrate (>98%), ammonium iron sulfate dodecahydrate (>98%), paraffin wax, kerosene, ethanol (>99.5%), and NaCl (>99.5%) were purchased from Aladdin Biochemical Technology Co., Ltd., Shanghai, China. Graphene oxide (GO) was supplied by Turing Evolution Technical Company, Shenzhen, China. Toluene (>99.5%) was acquired from Sinopharm Chemical Reagent Co., Ltd., Shanghai, China. Double-distilled water was used throughout the experiments.

### 3.2. Preparation of MJGO

**Figure 13:**
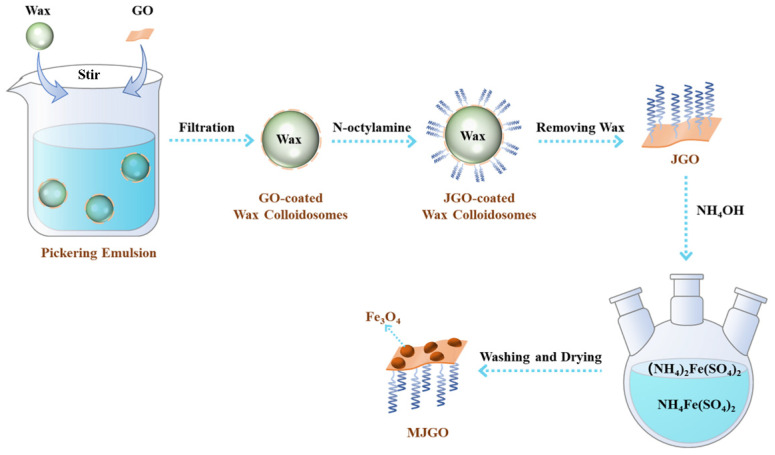
Schematic diagram of the MJGO synthetic process.

The MJGO was prepared by in situ chemical coprecipitation (Figure 13). The JGO was synthesized via the Pickering emulsion method as previously described [34]. Firstly, the mixture of 200 mL GO aqueous solution (1 mg/mL), 6 g NaCl, 50 g paraffin, and 100 g water was heated to 65 °C. Afterwards, the heated mixture was stirred using a FJ200-S homogenizer (Lichen Instrument Technology Co., Ltd., Shanghai, China) at the rate of 10,000 rpm to form the emulsion. After the emulsion was cooled to room temperature, GO-coated paraffin microspheres were obtained through suction filtration. Furthermore, single-layer GO-coated paraffin microspheres were obtained by further washing with water and ethanol. Then, the single-layer, GO-coated paraffin microspheres were added into the n-octylamine ethanol solution (0.4489 mg/mL) and magnetically stirred overnight to obtain the JGO-coated paraffin microspheres. The mixture of the JGO-coated paraffin microspheres and petroleum ether was heated to 65 °C to dissolve the paraffin. Then, the final JGO product was obtained after further centrifugation, washing, and drying. Then, 25 mg JGO was dispersed in 25 mL deionized water for 60 min by ultrasonic dispersion to obtain a uniformly and stably dispersed JGO solution. Then, 127.0 mg (NH_4_)_2_Fe(SO_4_)_2_·6H_2_O and 312.4 mg NH_4_Fe(SO_4_)_2_·12H_2_O were added into the JGO dispersion solution and dispersed by ultrasonic dispersion for 5 min to render them evenly dispersed. The reaction was set at 60 °C and under a nitrogen atmosphere. NH_4_·OH was added at a speed of 1 d/s until the pH was adjusted to around 11. Finally, the precipitation was separated by magnetic separation and then washed with deionized water and ethanol and vacuum freeze-dried for 12 h [37,38].

### 3.3. Characterization

The PerkinElmer Spectrum (VERTEX 70, Bruker, Billerica, MA, USA) was employed to record the Fourier-transform infrared spectra (FTIR) of Fe_3_O_4_, GO, JGO, and MJGO. A transmission electron microscopy (TEM) image of the MJGO was obtained with a JEOL JEM-1400 transmission electron microscope (JEOL, Tokyo, Japan). The X-ray diffraction (XRD) profiles of the Fe_3_O_4_, JGO, and MJGO were recorded using an XRD–7000 diffractometer (Shimadzu Corporation, Kyoto, Japan). The magnetic properties of the MJGO were investigated using the physical property measurement system (Lake Shore Cryotronics, 7400-S, Westerville, OH, USA) at 298 K. In addition, the water contact angles of the glass surfaces and waxed glass surfaces with undeposited MJGO and deposited MJGO were photographed using a DCA15 interface expansion rheometer.

### 3.4. Preparation of Crude Oil-in-Water Emulsion

The crude oil utilized in the investigation was sourced from Shengli oil field. The oil-in-water emulsion with an oil content of 2% *v*/*v* (2:98) was prepared by mixing NaCl aqueous solution and crude oil diluted with kerosene [39,40] and stirred at 10,000 rpm with a homogenizer for 20 min.

### 3.5. Emulsion Separation

The particle dispersion was injected into the emulsion, followed by violent shaking for 120 s. The separation process of the crude oil-in-water emulsion was recorded by the camera after allowing the mixture to stand. The MJGO nanoparticles were collected from the mixture using a magnet, and the residual oil present in the collected water was extracted by utilizing petroleum ether. The UV–vis spectrometer was employed to measure the absorbance of the oil at 254 nm. The absorbance of different concentrations of crude oil/petroleum ether solution was measured to obtain the standard curve of absorbance−oil content. The difference in absorbance between the water sample containing oil residue and pure water was calculated to determine the oil content using the standard curve. Then, the concentration of the oil residue was calculated by the following formula:

(3) $$C=\frac{m\times 1000}{V}$$

*C*, *m*, and *V* represent the oil residue content of the water sample (mg/L), the corresponding oil content in the standard curve (mg), and the volume of the water sample (mL), respectively.

Then, the corresponding separation efficiency of the MJGO was calculated via the following equation [41,42,43]:

(4) $$S_{e}=\frac{C_{0}-C_{1}}{C_{0}}\times 100\%$$

where *S_e_* (%) represents the separation efficiency, and *C*_0_ and *C*_1_ represent the oil content in the emulsion prior to and subsequent to the particle separation, respectively.

In addition, the absorption spectrum of the crude oil-in-water emulsion before and after separation was determined by the UV–vis spectrometer. An optical microscope (PH50-3A43L-A, Phenix, Shanghai, China) was employed to observe the morphological characteristics of the crude oil-in-water emulsion before and after separation. The zeta potentials of the emulsified oil droplets, JGO dispersion, and MJGO dispersion at different pH levels were measured by the Malvern Zetasizer Nano series (Malvern company, Malvern, UK). The emulsion concentration was diluted to 200 ppm prior to zeta potential measurement in order to ensure the accuracy of the analysis.

### 3.6. Recycle Tests

Following the emulsion separation, the MJGO nanoparticles were retrieved using a magnet and washed by toluene solution to eliminate any attached oil [44,45,46]. The recovered MJGO was continuously utilized for subsequent demulsification experiments.

## 4. Conclusions

In summary, we successfully fabricated a novel and recyclable demulsifier MJGO by introducing magnetic Fe_3_O_4_ to JGO. Compared with the GO and JGO, the MJGO displayed a superior demulsification efficiency as high as 96% in separating crude oil-in-water emulsion tests. The oil absorbance test showed that even 50 mg/L MJGO could also separate the crude oil and water effectively. Moreover, the acidic and natural conditions (pH ≤ 6) and high temperature were conducive to the MJGO demulsification performance. In our previous research, it was found that the strong interaction between JGO and an emulsion interfacial film caused the separation of crude oil and water. In the present study, the MJGO with a lower zeta potential exhibited weakened electrostatic repulsion with negatively charged oil droplets, which greatly promoted the adsorption capacity of the MJGO on the interface. Accordingly, the superior demulsification performance of MJGO compared to that of JGO should be attributed to its higher adsorption capacity on the interface, rendering the oil/water interface easier to deform. More importantly, the MJGO possesses a considerable demulsifying ability even after being reused seven times. This study provides a novel method to prepare a recyclable and high-performance GO-based demulsifier, which shows great potential in separating emulsions in the oil industry. Given the intricacy and unpredictability of the oilfield environment, it is imperative to conduct additional field trials and be equipped with state-of-the-art recovery equipment to ensure its effective and practical application.

## Figures and Tables

**Table 1:** Crystallographic data for MJGO.

Sample	2*θ*	Crystal Plane (h k l)	FWHM (°)	Crystal Size, D (nm)	d-Spacing (Å)
MJGO	30	(2 2 0)	0.32	26	0.29
	35	(3 1 1)	0.31	27	0.25
	43	(4 0 0)	0.39	22	0.21
	53	(4 2 2)	0.60	15	0.17
	57	(5 1 1)	0.49	18	0.16
	63	(5 3 3)	0.47	20	0.14

## Data Availability

The original contributions presented in the study are included in the article/Supplementary Material, further inquiries can be directed to the corresponding author/s.

## Supplementary Materials

The following supporting information can be downloaded at the following link: https://www.mdpi.com/article/10.3390/molecules29143307/s1, Figure S1: SEM image of MJGO nanostructure; Figure S2: The crude oil-in-water emulsion of freshly prepared, ageing for 24 h and ageing for 72 h; Figure S3: Photographs and particle size analysis of the crude oil-in-water emulsion before and after separation by MJGO particles; Figure S4: The zeta potential of MJGO under different numbers of cycle.
