# Supplementary material for: Facile Synthesis of Novel Magnetic Janus Graphene Oxide for Efficient and Recyclable Demulsification of Crude Oil-in-Water Emulsion
Source: Molecules. 2024 Jul 13;29(14):3307. doi: 10.3390/molecules29143307 (PMC11279671; doi:10.3390/molecules29143307)
Supplement: Supplementary file 1 [file molecules-29-03307-s001.zip › molecules-3028153-supplementary.pdf]

Supporting Information for

Facile Synthesis of Novel Magnetic Janus Graphene Oxide for Efficient and Recyclable Demulsification of Crude Oil-in-Water Emulsion

Yingbiao Xu [1,2], Li Cheng [3], Yefei Wang [1,*] and Han Jia [1]

[1] *Key Laboratory of Unconventional Oil & Gas Development, China University of Petroleum (East China), Ministry of Education, Qingdao 266580, China.*

[2] *Technology Inspection Center, Shengli Oilfield Company, SINOPEC, Dongying 257000, China.*

[3] *School of Petroleum Engineering, Yangtze university, Wuhan 430100, PR China.*

Corresponding authors:

Yefei Wang, Tel.: 0532-86981702, E-mail address: wangyf@upc.edu.cn.

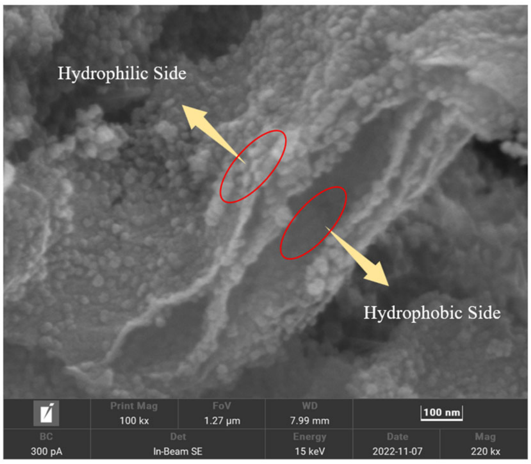

Figure S1. SEM image of MJGO nanostructure.

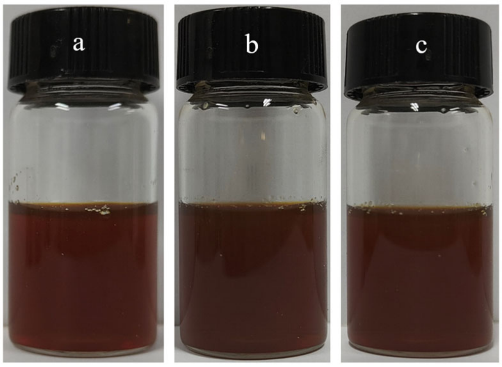

Figure S2. The crude oil-in-water emulsion of (a) freshly prepared, (b) ageing for 24 h and (c) ageing for 72 h.

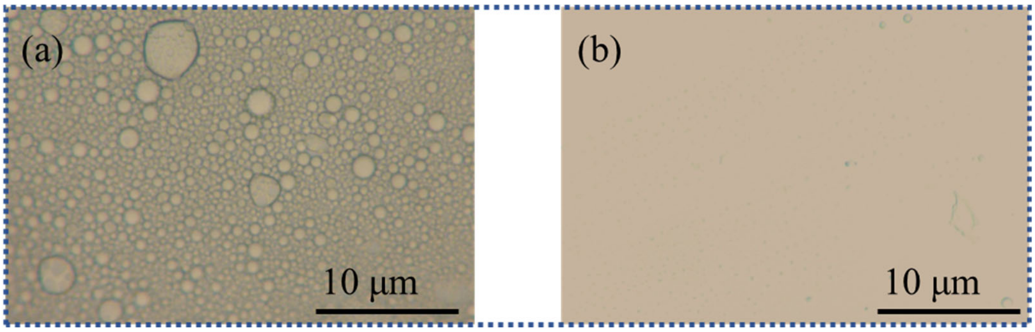

Figure S3. Photographs and particle size analysis of the crude oil-in-water emulsion before (a) and after (b) separation by MJGO particles.

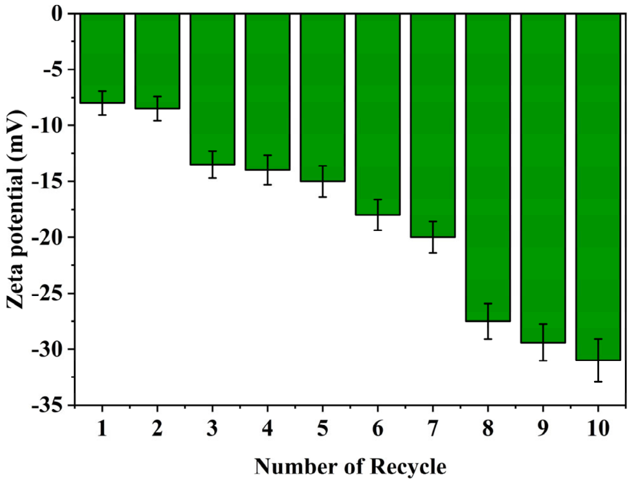

Figure S4. The zeta potential of MJGO under different numbers of cycle.
